# A Proof-of-Concept Study for the Efficacy of Dispirotripiperazine PDSTP in a Mouse Model of Herpes Simplex Herpetic Encephalitis

**DOI:** 10.3390/v18090944

**Published:** 2026-08-28

**Authors:** Kristina Pomeshikova, Olga Riabova, Natalia Monakhova, Irina Leneva, Giuseppina Sanna, Vadim Makarov

**Affiliations:** 1 Federal Research Centre “Fundamentals of Biotechnology” of the Russian Academy of Sciences (Research Centre of Biotechnology RAS), 33-2 Leninsky Prospect, Moscow 119071, Russia; k.pomeshikova@fbras.ru (K.P.); o.riabova@fbras.ru (O.R.); n.monakhova@fbrsa.ru (N.M.); 2 I.M. Sechenov First Moscow State Medical University, Moscow 119991, Russia; 3 Department of Virology, Mechnikov Research Institute of Vaccines and Sera, Moscow 105064, Russia; wnyfd385@yandex.ru; 4 Microbiology and Virology Unit, Department of Biomedical Science, University of Cagliari, 09042 Monserrato, Italy; g.sanna@unica.it

**Keywords:** PDSTP, herpes simplex virus type 1, preclinical efficacy, herpetic encephalitis, antiviral drug

## Abstract

Herpes simplex encephalitis (HSE) remains a life-threatening disease with high mortality and severe neurological complications, despite acyclovir therapy being limited by delayed diagnosis, drug resistance, and poor blood–brain barrier penetration. New antivirals with different mechanisms of action are urgently needed. We evaluated PDSTP, a novel dispirotripiperazine-based compound targeting host heparan sulfate proteoglycans (HSPGs), in mouse models of HSV-1 encephalitis. This study evaluates the therapeutic and prophylactic potential of PDSTP, an antiviral compound that blocks HSV glycoconjugates on the host cell membrane, using a well-established mouse model of herpes encephalitis. BALB/c mice were infected with HSV-1 strains VR-539 or VR-733 via intraperitoneal (IP) or intranasal (IN) routes. PDSTP was administered orally (PO) or intraperitoneally (IP) at 12.5–80 mg/kg/day, alone or in combination with acyclovir (10–100 mg/kg/day), using a prophylactic–therapeutic regimen (2 h pre-infection, then twice daily for 7 days). Survival, weight loss, mean survival time, and brain viral loads were assessed. PDSTP at 80 mg/kg/day (PO) protected 30–40% of VR-733-infected mice and extended lifespan 1.9-fold (*p* < 0.05), comparable to acyclovir at 100 mg/kg/day. IP administration was superior to PO. The combination of IP PDSTP (12.5 mg/kg/day) with IP acyclovir (10 mg/kg/day) achieved 100% survival, prevented weight loss, and significantly reduced brain viral titers (*p* < 0.001) in both infection models. PDSTP demonstrates dose- and strain-dependent activity as monotherapy and enhanced efficacy when combined with acyclovir. The IP combination provided complete protection at low doses, supporting PDSTP as a promising candidate for further development against HSE.

## 1. Introduction

HSE is the most common sporadic viral encephalitis with fatal outcome worldwide [1,2]. Despite the fact that acyclovir was introduced more than four decades ago, mortality remains at about 20%, and most survivors suffer from permanent neurological deficits [3,4]. Clinical problems include a narrow therapeutic window, the emergence of drug-resistant herpes virus strains, and insufficient drug penetration into the central nervous system (CNS). These limitations highlight the urgent need for new antiviral drugs with different mechanisms of action.

**Figure 1:**
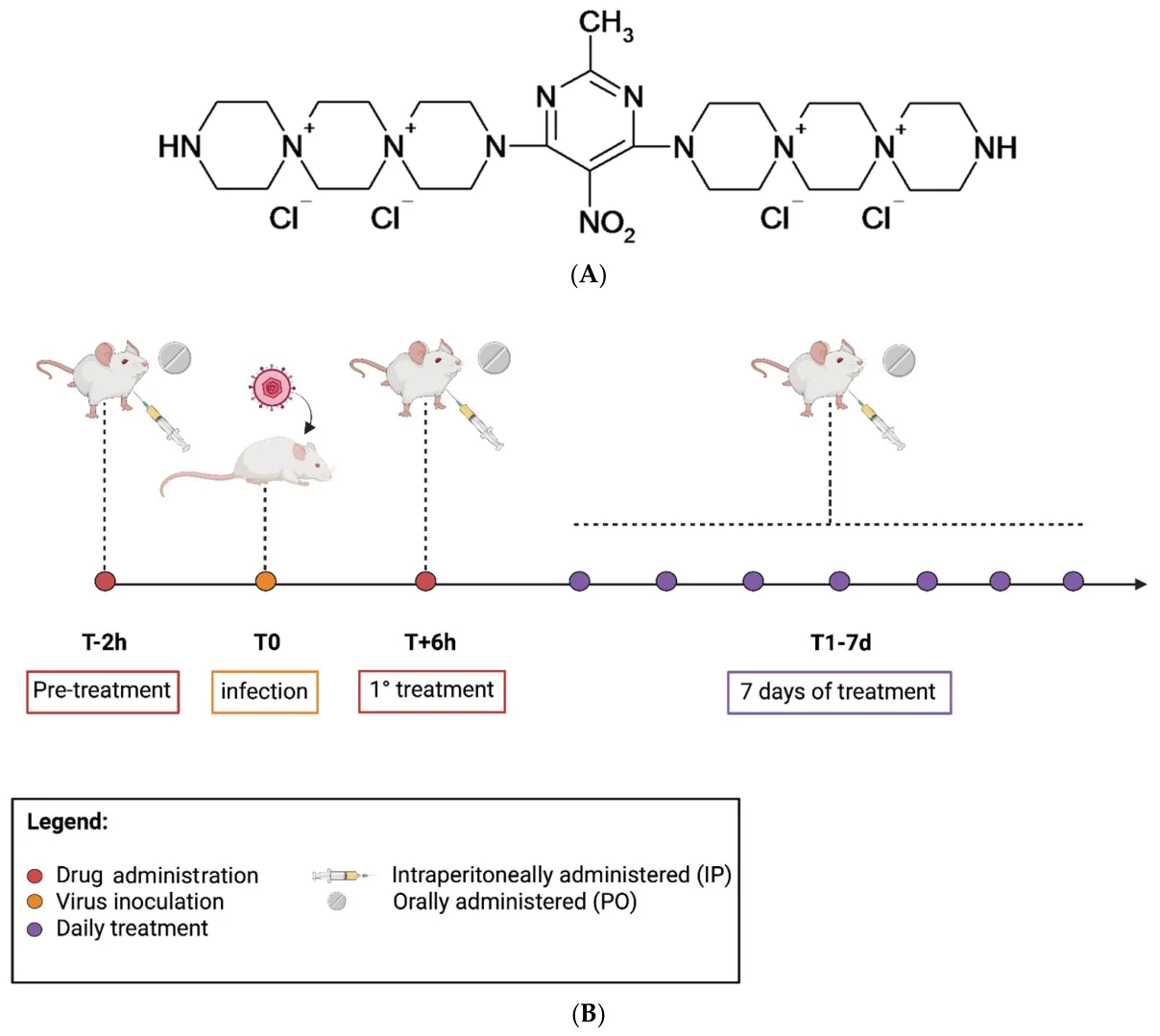
(**A**) Chemical structure of PDSTP. (**B**) Scheme of in vivo efficacy study design. PDSTP (**A**), a dispiro compound with two shared quaternary nitrogen atoms, demonstrates potent in vitro antiviral activity against various viruses, including herpes simplex viruses 1 and 2, papillomaviruses, cytomegalovirus, and respiratory syncytial virus [5,6,7,8,9]. These viruses share heparan sulfate proteoglycans (HSPG) as a cellular receptor for attachment and entry, which PDSTP blocks by binding to HSPG [10,11,12]. DSTP-based compounds, including PDSTP and DSTP27, inhibit viral adsorption by interacting with HSPG, showing high antiviral activity against HSV-1/2 (IC_50_ 1.48–20.4 µM), HIV (IC_50_ 1.4–150 µM), HPV (IC_50_ 0.7–2.6 µM), and CMV (IC_50_ 0.15–0.85 µM) [13]. The 5-nitropyrimidine fragment enhances their antiviral efficacy. PDSTP also exhibits triple antibacterial activity, including blocking adhesion, enhancing antibiotics, and preventing biofilms, particularly against *P. aeruginosa* [14]. The data obtained demonstrate the promising use of the compound for the preventive–therapeutic of encephalitis caused by the HSV-1 [15,16,17,18].

To assess the preventive and therapeutic efficacy of the compounds, a schedule for drug administration was developed (Figure 1B). The first dose was administered 2 h before infection (T − 2h) in order to reach a therapeutic level by the time of contact with the pathogen. The viral infection was carried out at time zero (T0). The second dose was administered 6 h after infection (T + 6 h) to simulate early preventive–therapeutic regimen Daily therapy was performed from day 1 to day 7 (T1 − 7d). The therapy was performed twice a day at 9:00 and 19:00 to maintain a stable concentration of the drug. The solution of the substances and acyclovir was prepared in such a way that the calculated dose of the drug was contained in a volume of 200 µL. A single-use insulin syringe with a special needle (lavage) was used for oral administration, and the suspension was injected intragastrically into mice in a volume of 200 µL. The doses were calculated in relative weight units—mg/kg of animal body weight. A control group was included in the study. Acyclovir served as a reference control for comparative analysis.

Acyclovir (9-(2-hydroxyethoxymethyl guanine)) is a synthetic antiviral drug used to treat herpes viruses, including HSV-1, HSV-2, varicella zoster, and Epstein–Barr virus. It is effective against genital and labial herpes, shingles, chickenpox, and herpetic encephalitis. Early use, especially during the prodromal stage, is very effective. Intravenous acyclovir reduces mortality from 33% to 22% in ventilated patients with HSV reactivation but does not affect ventilation duration. Oral acyclovir has a bioavailability of approximately 20%, with a maximum concentration of 0.7 μg/mL from 200 mg tablets and 20.7 μg/mL intravenously (10 mg/kg). Its half-life is 2.5–3.3 h PO extending to 19.5 h with renal insufficiency. Acyclovir is primarily excreted unchanged via the kidneys (45–79%). Advantages include high selectivity, low toxicity, and multiple dosage forms. Disadvantages include low oral bioavailability, potential resistance in immunocompromised patients, and side effects like nephrotoxicity, headache, and nausea [19,20,21,22].

In this study, acyclovir was selected as the primary comparator for several reasons. First, it is the first-line therapy for HSE, the most severe manifestation of HSV-1 infection that this study models. Second, its mechanism of action (inhibition of viral DNA polymerase) is fundamentally different from that of PDSTP (viral entry inhibition via HSPG binding), allowing us to explore the potential for synergistic interaction between two distinct antiviral strategies. While prodrugs like valacyclovir offer improved pharmacokinetics, they are ultimately metabolized to acyclovir and share its mechanism of action and resistance profile. Therefore, the direct comparison with the parent compound acyclovir provides the clearest assessment of the intrinsic efficacy and complementary potential of PDSTP. It is important to note that in this study we did not test PDSTP on HSV strains resistant to acyclovir.

Previously, we published an article demonstrating the antiherpetic efficacy of PDSTP in a mouse model of herpetic encephalitis with intraperitoneal and oral administration both as monotherapy and in combination with acyclovir [23]. In this study, PDSTP monotherapy had no significant effect—only the combination with acyclovir was effective. Combination therapy with intraperitoneal administration demonstrated pronounced synergism; however, with oral administration, the effect was moderate. This work is the first confirmation of PDSTP activity in vivo. The published study contains a number of significant shortcomings and omissions, such as the use of untested virus strains, errors in statistics, and so on, so it was necessary to conduct a new scientifically based study that would eliminate these errors. This work is a logical continuation of the previous study, in which we eliminated these limitations.

## 2. Materials and Methods

### 2.1. Biosafety and Ethics Statement

All experiments with the HSV-1 and models of herpetic encephalitis were conducted in accordance with established biosafety standards and ethical standards. The work used specially equipped laboratory facilities in compliance with the requirements of biosafety level 2, which ensured the safety of personnel and the prevention of the spread of the virus. All live experiments on mice were performed in accordance with protocols approved by the Ethical Committee of the Institute of Therapy and Immunology and complied with national and international standards for the humane preventive–therapeutic of animals, namely: the Rules on Ethics of conducting research on animals, current regulations of the Russian Federation, as well as the requirements of the NIH manual (Guide for the Care and Use of Laboratory Animals, 8th edition, 2011). Before the start of the study, all necessary permits were obtained to ensure compliance with bioethical requirements and animal welfare standards.

### 2.2. Efficacy Study

All studies with animals were approved by the Mechnikov Research Institute of Vaccines and Sera Committee on the Ethics of Animal Experiments and were conducted in strict accordance with the applicable laws and guidelines (Study number 13/2025).

### 2.3. Cell Lines

African Green monkey kidney (Vero) cell line was purchased from the American Type Culture Collection (ATCC), Manassas, VA, USA. The cells were cultured in the growth medium consisting of DMEM (Dulbecco’s modified Eagle medium, PanEco, Moscow, Russia) supplemented with 10% inactivated fetal bovine serum (FBS, PanEco, Russia), 2 mM L-glutamine (Sigma-Aldrich, St. Louis, MO, USA) and antibiotics 100 units/mL of penicillin and 100 μg/mL of streptomycin and 40 μg/mL of gentamycin (PanEco, Russia). The cells were incubated in 5% CO_2_ atmosphere at 37 °C.

### 2.4. Virus Strains

Herpes simplex virus type 1 strains VR-733 and VR-539 purchased from the ATCC distributor. HSV-1 strain VR-733 was isolated from the patient’s facial vesicle and is commonly used in virus susceptibility testing, while strain VR-539 was isolated from the brain of a human with encephalitis and is commonly used in infectious disease research. These strains are characterized by stable replication ability, different tropicity to target cells, and different neurovirulence, which was previously established experimentally. We selected these strains for the experiment in order to obtain the most complete characterization of PDSTP activity. The virus was propagated on Vero cells and stored at a temperature of −70 °C. Virus titers were determined using the standard endpoint dilution method, as described earlier [24]. Virus titers were evaluated using a standard method (Reed and Muench), a micromethod in 96-well culture plates using cell culture and expressed in lg TCID_50_/mL. In most experiments, viruses were used in the form of a virus-containing suspension with an infectious titer of 6.0 lg TCID_50_/mL. All the obtained viruses were stored frozen in aliquots.

### 2.5. Compounds and Chemicals

PDSTP (3,3′-(2-methyl-5-nitropyrimidine-4,6-diyl)bis-3,12-diaza-6,9-diazonia-dispiro(5.2.5.2)hexadecane tetrachloride dihydrochloride nonahydrate) was synthesized as described previously [7]. Acyclovir (9-(2-hydroxyethoxymethyl) guanine) (the drug in ampoules, Zovirax, Glaxo Smith Klein, Warsaw, Poland) was used as a comparison drug.

### 2.6. Animals

Female BALB/c mice weighing 12–14 g were obtained from the Andreevka nursery of the Russian Academy of Medical Sciences (Moscow Region) and kept on a standard diet under regulated vivarium conditions. The animals were kept in accordance with the rules for the establishment, equipment and maintenance of experimental biological clinics (vivariums). The animals were fed with briquetted feed in accordance with approved standards. The animals were labeled by groups using dyes (eosin) on the body surface. The studies were conducted in accordance with the ethical regulatory documents in force in the Russian Federation (1–4), after approval by the Ethics Committee of the I.I. Mechnikov Federal State Budgetary Research Institute, as well as taking into account the recommendations of international licensing authorities. The choice of female mice is determined by the need to ensure reproducibility of the results, minimize data variability, and comply with generally accepted modeling protocols for herpetic encephalitis.

### 2.7. Evaluation of the Effectiveness of the Substance in a Mouse Model of Herpetic Encephalitis

The mouse model of herpetic encephalitis was reproduced in mice with two methods of infection. In preliminary experiments, a dose of the virus was determined for each method of infection, causing 100% death in the 10 × LD_50_ viral control group, which was then used in experiments to evaluate the effectiveness of the substance.

Intraperitoneal infection: BALB/c mice (females, average weight 12–16 g) were infected with the virus using a sterile disposable syringe with a 25 g needle, injecting the virus in a volume of 100 µL. After that, the animals were put back in their cages.

Intranasal infection: BALB/c mice (females, average weight 12–16 g) under light anesthesia were infected IN in each nostril with a suspension of HSV-1 or HSV-2 type using 20 µL automatic pipettes with sterile tips. After that, the animals were placed back in their cages until they fully recovered from anesthesia [25]. Intranasal infection of BALB/c mice with HSV virus was used as a model of herpetic encephalitis, which is a standard and verified model widely used in preclinical studies to evaluate the antiviral activity of compounds and study the pathogenesis of HSV infection [26,27,28]. This model reproduces the clinical path of development of herpetic encephalitis through the penetration of the virus through the olfactory tract, followed by damage to the central nervous system.

The preventive–therapeutic was performed both PO and IP. With both routes of administration, the following preventive–therapeutic regimen was used: 2 h before infection, then 6 h later, then for the next 7 days 2 times a day with a break of 10 h. The preventive–therapeutic was usually performed at 9 a.m. and 7 p.m. The solution of the substances and acyclovir was prepared in such a way that the calculated dose of the drug was contained in a volume of 200 µL. A single-use insulin syringe with a special needle (lavage) was used for oral administration, and the suspension was injected intragastrically into mice in a volume of 200 µL. The doses were calculated in relative weight units—mg/kg of animal body weight. There were 10 animals in each group. In some groups, 1–2 mice died when infected under anesthesia, as well as on the first and second days of preventive–therapeutic, so in the future such groups were considered as containing 9 and 8 animals, respectively. The treated and control animals were monitored daily for 21 days. In the first 5 days after infection, the mice were weighed every day, then every other day. The effectiveness of the chemotherapeutic activity of the compounds was evaluated according to three criteria: the indicator of protection against a deadly viral infection, an increase in average life expectancy, a decrease in weight loss in groups of animals treated with the drug compared with the control group, and a decrease in the titer of the virus in brain tissue. The average life expectancy of mice was calculated using the formula MSD = ∑f(d − 1)/*n*, where f is the number of mice that died on day d, the surviving mice are also included in f, and d in this case is 21, n is the number of mice in the group. Weight loss or increase was calculated separately for each mouse and expressed as a percentage. At the same time, the weight of the animal before infection was taken as 100%. The average percentage of weight loss or gain was determined for all mice in the same group. As part of experiments using a mouse model of herpetic encephalitis, three mice were slaughtered on the 5th day after infection in each study group and their brains were extracted under sterile conditions. After washing three times in phosphate–salt buffer (PSB) solution, the brains were homogenized in a Dounce homogenizer for 25 strokes and resuspended in 1 mL of cold sterile PSB solution. The suspension was cleaned of cell residues at 2000 g for 10 min, and then the supernatant was selected for further analysis.

### 2.8. Statistical Methods and Software Used

To determine the statistical difference in the dynamics of animal weight, the nonparametric Kruskal–Wallis test with the Bonferroni correction was used, at the significance level *p* < 0.05. Kaplan–Meyer curves were used to visualize survival data. The normality of the data distribution was assessed using the Shapiro–Wilk test. All graphs were plotted using the GraphPad Prism 10 program. The virus titer was calculated using the Reed–Muench method. The descriptive statistics parameters included the average value of the indicator in the group and the standard deviation.

## 3. Results

### 3.1. In Vivo Effectiveness of the Pyrimidine-Dispirotripiperazinium in a Mouse Encephalitis Model Caused by Intraperitoneal HSV-1 Infection

**Figure 2:**
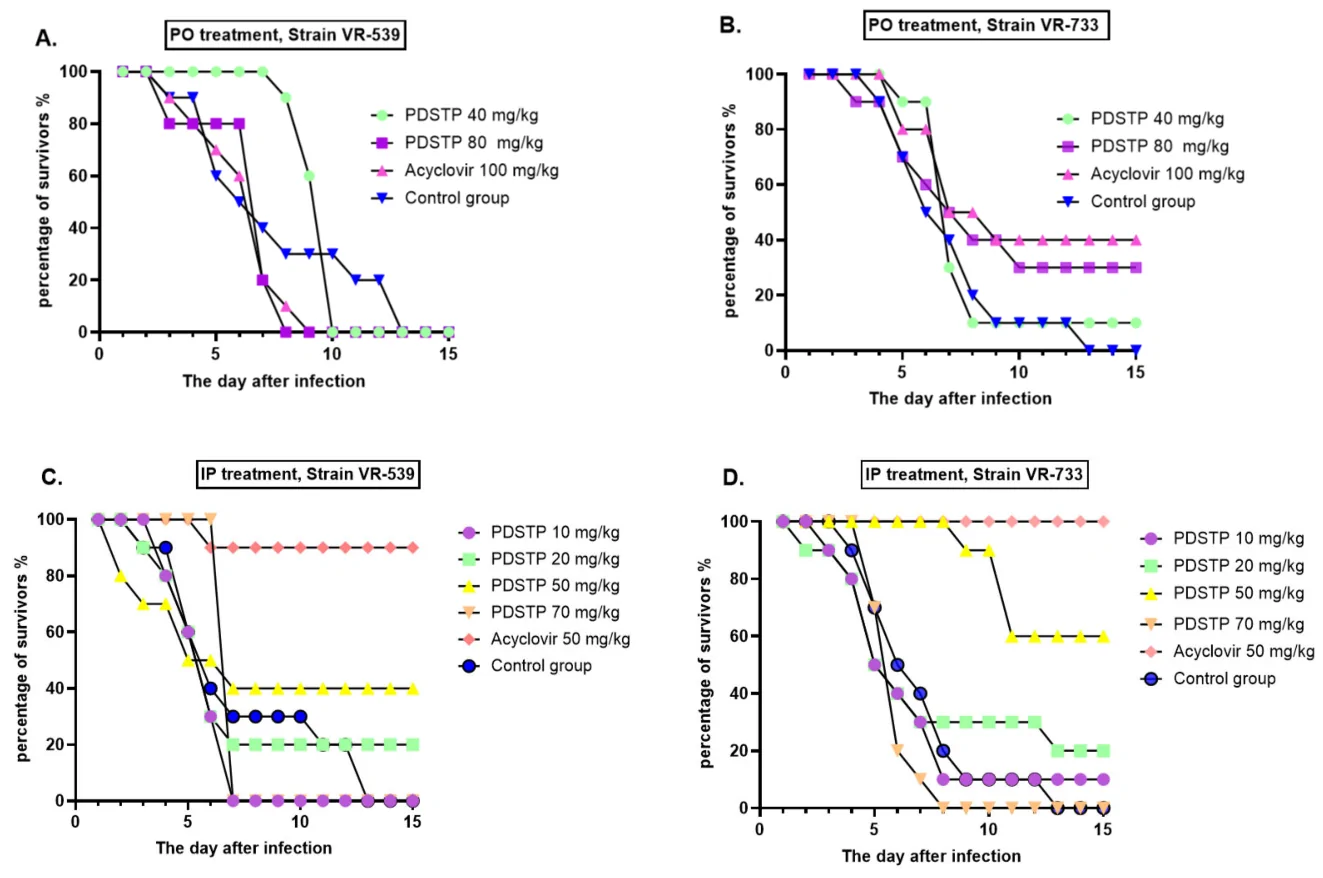
The effectiveness of the PDSTP on a model of herpetic encephalitis in mice with IP infection with HSV-1 strain VR-539 during PO and IP preventive–therapeutic. ((**A**) The effect of PO with PDSTP and acyclovir on the survival of mice with IP infection with HSV-1 strain VR-539. (**B**) The effect of PO with PDSTP and acyclovir on the survival of mice after IP infection with HSV-1 strain VR-733. (**C**) The effect of IP preventive–therapeutic with PDSTP and acyclovir on the survival of mice with IP infection with HSV-1 strain VR-539. (**D**) The effect of IP preventive–therapeutic with PDSTP and acyclovir on the survival of mice after IP infection with HSV-1 strain VR-733.).

PDSTP efficacy was evaluated using a rigorous lethal mouse encephalitis model with IP HSV-1 infection, causing acute systemic disease with CNS damage and viral. The compound was administered PO and IP using a therapeutic–prophylactic regimen: 2 h pre-infection, 6 h post-infection, then twice daily for 7 days. As a result of the research conducted, the data shown in Supplementary Materials Table S1, Figure 2, and summarized below were obtained.

In the IP infection model, PO administration of PDSTP at doses of 40 and 80 mg/kg/day, as well as acyclovir at 100 mg/kg/day, was ineffective in mice infected with HSV-1 strain VR-539. This preventive–therapeutic did not affect the survival of animals, did not increase the average life expectancy, and did not reduce weight loss compared with the viral control group. At a dose of 40 mg/kg/day, oral preventive–therapeutic of mice infected with another strain of HSV-1 VR-733 was also ineffective; however, an increase in the dose to 80 mg/kg/day with the same preventive–therapeutic method and the same regimen was comparable to preventive–therapeutic with acyclovir at a dose of 100 mg/kg/day. In these experiments, the preventive–therapeutic protected 40% of the animals, increasing their average life expectancy by 1.8–1.9 times (Figure 2A,B, SM additional figure to Table S1).

In Figure 2C,D, when administered intraperitoneally to strains VR-539 and VR-733, PDSTP demonstrated a pronounced dose-dependent antiviral effect in the range of 10–50 mg/kg: survival increased from 10–20% to 50–70%, respectively. The maximum efficacy was achieved at a dose of 50 mg/kg. With an increase in the dose to 70 mg/kg, a paradoxical decrease in survival (up to 10–20%) was observed in both strains, which is reproduced in four independent experiments on different strains and suggests the toxic effect of high concentrations of PDSTP on the background of virus-induced inflammation.

### 3.2. In the Second Series of Experiments, the Combined Effect of PDSTP in Low Doses and Acyclovir Was Studied

**Figure 3:**
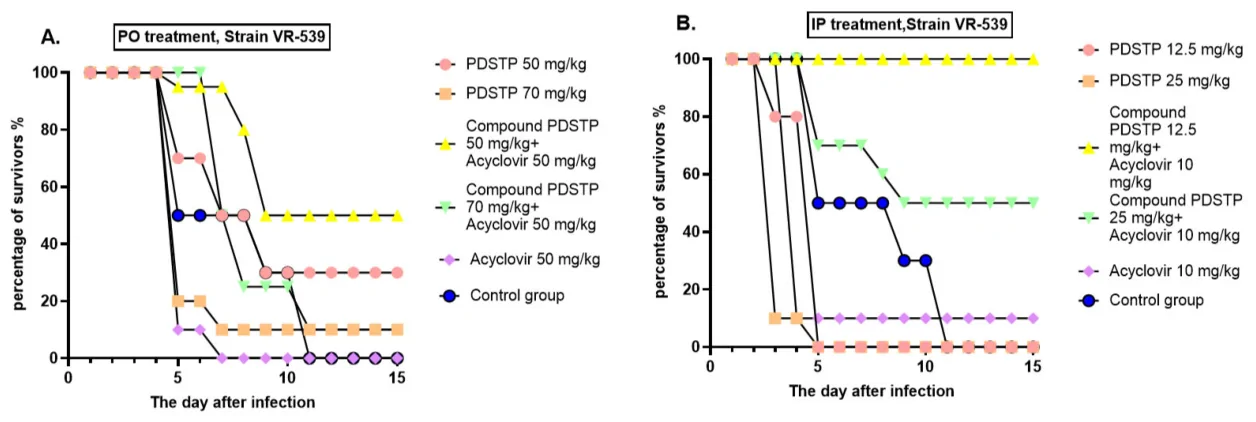
The effect of preventive–therapeutic with PDSTP alone and in combination with acyclovir on the survival of mice infected with lP HSV-1 ((**A**) Effect of PO preventive–therapeutic of the PDSTP substance alone and in combination with acyclovir on the survival of mice infected IP with HSV-1 strain VR-539. (**B**) The effect of IP preventive–therapeutic with the substance PDSTP alone and in combination with acyclovir on the survival of mice after IP infection with the HSV-1 strain VR-539.).

In the IP infection model, PO preventive–therapeutic of PDSTP (50 and 70 mg/kg/day) and acyclovir (50 mg/kg/day) were tested. When infected with HSV-1 strain VR-539, causing 100% mortality in controls, PDSTP at 70 mg/kg/day and acyclovir at 50 mg/kg/day alone were ineffective. PDSTP at 50 mg/kg/day protected 33% of animals, extending their lifespan by 1.3–1.4 times. Combining PDSTP at 50 mg/kg/day with acyclovir at 50 mg/kg/day was more effective, preventing death in 55% of animals, reducing weight loss, and doubling lifespan. However, combining PDSTP at 70 mg/kg/day with acyclovir at 50 mg/kg/day showed no significant improvement over individual preventive–therapeutics (Figure 3A). IP preventive–therapeutic of PDSTP 12.5 mg/kg/day in combination with acyclovir at a dose of 10 mg/kg/day was highly effective and completely prevented animal mortality and weight loss (Figure 3B). Increasing the PDSTP dose to 25 mg/kg/day in combination with acyclovir at a dose of 10 mg/kg/day provided partial protection survival rate of 50%, 12.7 days (Figure 3, SM additional figure to Table S2).

### 3.3. In Vivo Effectiveness of the Pyrimidine-Dispirotripiperazinium Substance in a Mouse Encephalitis Model Caused by Intranasal HSV-1 Infection

The next stage of the study examined the efficacy of PDSTP in a mouse HSV-1 encephalitis model. Mice were IN infected with HSV-1 strains VR-539 at 10 × LD_50_ doses. PO and IP preventive–therapeutics were administered 2 h before infection, followed by 6 h later, and daily for 7 days with a 10 h break. Acyclovir served as a reference drug.

**Figure 4:**
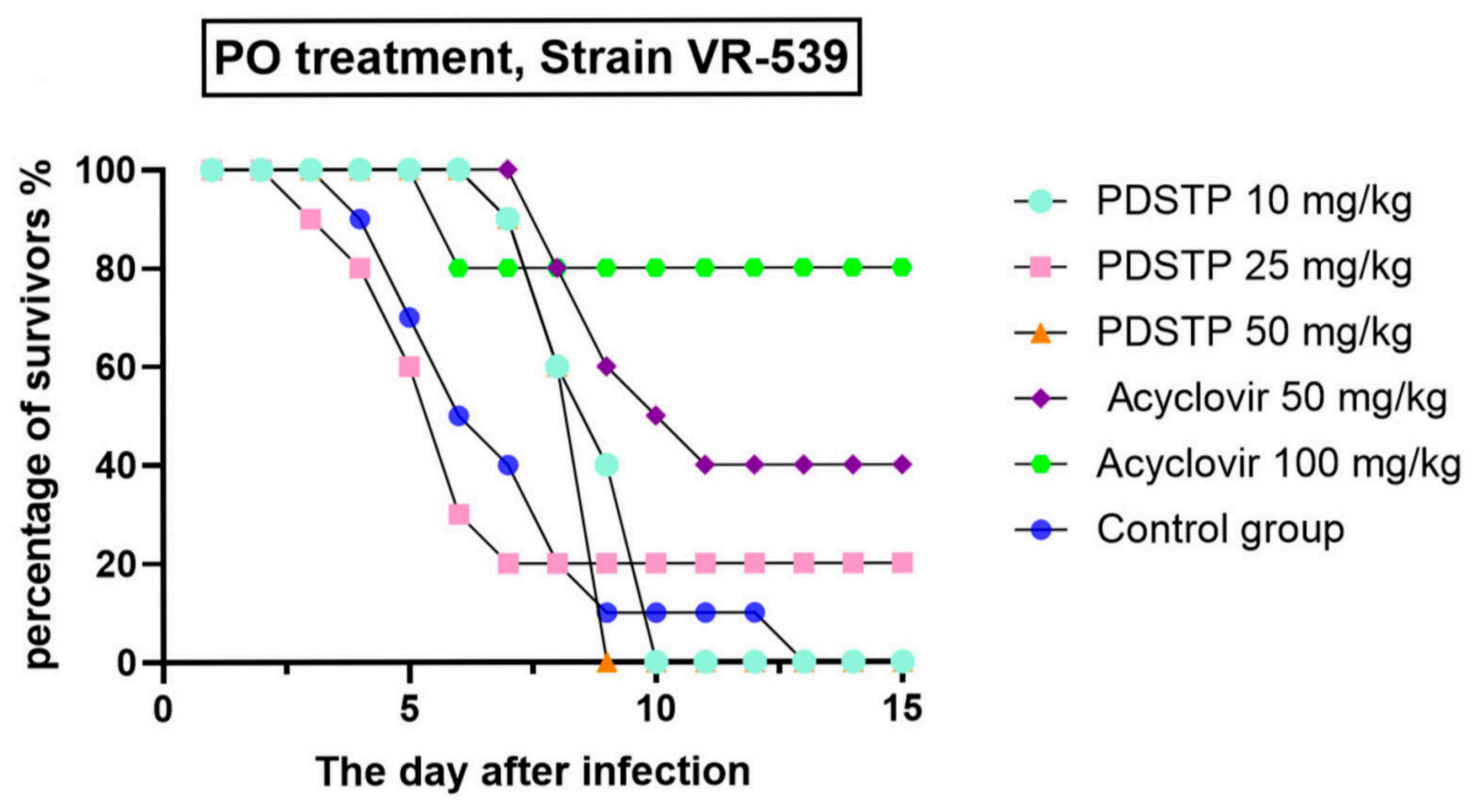
Efficacy of PO with PDSTP substance in mouse encephalitis induced by intranasal infection with HSV-1 strain VR-539 (Efficacy of the PDSTP substance on a mouse model of herpetic encephalitis in case of IN infection with HSV-1 strain VR-539 during PO).

For the HSV-1 strain VR-539 untreated mice died within 5.5 days. Oral PDSTP at 10 and 50 mg/kg/day was ineffective, while 25 mg/kg/day protected 20% of mice, increasing their life expectancy 1.6-fold. Oral acyclovir at 50 and 100 mg/kg/day was highly effective, protecting 40% and 80% of mice with life expectancy increases of 2.2- and 3.2-fold, respectively (Figure 4, Supplementary Materials Table S3). Thus, in intranasal infection, PDSTP within the studied dose range (10–50 mg/kg) did not provide significant protection to the animals, which indicates that the dose was insufficient to achieve a therapeutic effect in this model. These data served as the basis for using higher doses of PDSTP in subsequent experiments.

**Figure 5:**
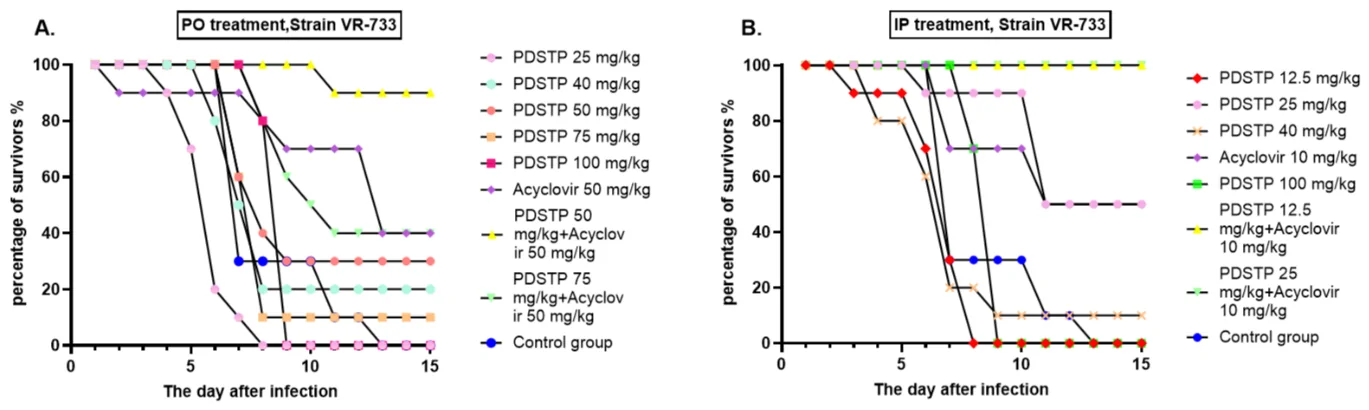
Efficacy of PO and IP preventive–therapeutic with PDSTP substance in mouse encephalitis induced by intranasal infection with HSV-1 strain VR-733 ((**A**) The effect of PO preventive–therapeutic with PDSTP alone and in combination with acyclovir on the survival of mice after IN infection with HSV-1 strain VR-733. (**B**) The effect of IP preventive–therapeutic with PDSTP alone and in combination with acyclovir on the survival of mice after IN infection with HSV-1 strain VR-733.).

In the next series of experiments, the VR-733 strain was used for IN infection. According to the dose of infection, 10 out of 10 mice infected with the virus and not treated with the drug (viral control) died on the 13th day of observation. The average life expectancy in the viral control group was 7.4 days. Figure 5 and Table S4 present similar results for the HSV-1 strain VR-733, showing the effects of PDSTP PO and IP preventive–therapeutics.

PDSTP PO preventive–therapeutic (Figure 5A) varied by dose: 40 and 50 mg/kg/day protected 20% and 30% of mice, extending life expectancy by 1.2 and 1.4 times, respectively. However, combining acyclovir with the highest PDSTP dose (75 mg/kg/day) did not improve outcomes over acyclovir alone. IP PDSTP preventive–therapeutic was also tested (Figure 5B). Doses of 12.5 and 40 mg/kg/day were ineffective, while 25 mg/kg/day equaled acyclovir at 10 mg/kg/day: 50% survival, 1.8–2 times longer life expectancy. Combining PDSTP (12.5 or 25 mg/kg/day) with acyclovir (10 mg/kg/day) was highly effective: no deaths, no weight loss, and complete protection (Figure 5, Supplementary Materials Table S4).

Complete protection and suppression of virus replication in the brain were demonstrated in the intranasal VR-733 model.

### 3.4. Brain HSV-1 Titers Following Oral/IP Preventive–Therapeutic with PDSTP Alone or in Combination with Acyclovir in Intraperitoneally Infected Mice

Data on drug effectiveness, assessed by mortality, average life expectancy, and weight loss, were confirmed by virological studies. Brain virus titers were highest in untreated controls (3.55 × 10^4^ to 1.29 × 10^5^ TCID_50_ with some animals showing lower titers of 1.0 × 10^3^ to 1.0 × 10^4^ TCID_50_ as detailed in Table S5). All preventive–therapeutics reduced brain viral titers, but the extent varied. Low-dose PDSTP (PO/IP) had no statistically significant effect. Increasing PDSTP dose significantly lowered viral titers compared to controls, but not more than acyclovir alone. The PDSTP–acyclovir combination showed the strongest viral titer reduction, significantly lowering brain viral titers compared to all other preventive–therapeutics and controls (Table S5). The graph clearly shows that the new PDSTP substance is a promising antiviral agent that exhibits additive effect with acyclovir. The most effective preventive–therapeutic regimen in this experiment was a combination of acyclovir and a low dose of PDSTP administered intraperitoneally. This paves the way for the creation of new combination drugs for the preventive–therapeutic of herpesvirus infections, potentially reducing the dose of each drug and reducing the risk of side effects.

### 3.5. Brain HSV-1 VR-733 Titers Following Oral/IP Preventive–Therapeutic with PDSTP Alone or in Combination with Acyclovir in Intranasally Infected Mice

Determination of the viral titer in the animal brains showed that in the control group of untreated animals, it was 5.75 × 10^1^ to 7.94 × 10^2^ TCID_50_ with some animals showing lower titers (down to 7.41 × 10^0^ to 2.82 × 10^1^ TCID_50_), as detailed in Table S6, which was significantly lower than in control groups of animals that were infected intraperitoneally. According to Table S6, the preventive–therapeutic with the PDSTP at a dose of 25 mg/kg/day intraperitoneally, acyclovir in all investigated cases, and their combinations, significantly lowered brain viral titers in mice. The analysis of viral load confirmed the high efficiency of therapy in this model. The key result of this stage of the experiment was that the combined IP administration of PDSTP and acyclovir not only completely prevented the death of animals but also led to the complete suppression of viral replication in the brain, which makes this scheme the most promising for further preclinical studies, thus explaining the enhanced efficacy of the PDSTP–acyclovir combination.

## 4. Discussion

This proof-of-concept study provides in vivo evaluation of PDSTP, a novel dispirotripiperazine-based compound, in two murine models of HSV-1 encephalitis—intraperitoneal (IP) and intranasal (IN) viral inoculation—with PDSTP and acyclovir administered via oral (PO) and intraperitoneal (IP) routes. The aim of this study was to demonstrate the potential for treating an HSV-induced disease using a well-established therapeutic and prophylactic model with an antiviral compound that blocks HSV glycoconjugates on the host cell membrane. Our findings demonstrate that PDSTP exhibits dose-dependent antiviral activity as monotherapy and, more importantly, that its combination with acyclovir yields substantially enhanced therapeutic outcomes, including complete protection and brain viral clearance in the IN infection model. The mechanism underlying PDSTP’s antiviral activity is well established at the molecular level. Members of the dispirotripiperazine (DSTP) family, including PDSTP and its analog DSTP-27, inhibit viral replication by blocking viral attachment to host cell surface heparan sulfate structures that serve as initial attachment receptors for many enveloped viruses [16,17]. This host-directed mechanism is particularly attractive because it imposes a high barrier to the development of viral resistance—a growing clinical concern with nucleoside analogs like acyclovir. Although we did not test PDSTP against acyclovir-resistant strains in this study, previous studies with DSTP-27 have shown activity against such strains in vitro [17]. This remains to be confirmed for PDSTP. The enhanced efficacy of the PDSTP–acyclovir combination observed in our study is mechanistically plausible and aligns with previous findings for dispirotripiperazine derivatives [13] which reported that combination therapy with a dispirotripiperazine compound and acyclovir in a mouse model of herpetic encephalitis resulted in greater protection than either agent alone, with brain viral titers significantly reduced compared to monotherapy groups. Efficacy of PDSTP monotherapy in the IP infection model: Oral PDSTP at 40 and 80 mg/kg/day showed no significant protection against strain VR-539 (0% survival; *p* > 0.05 vs. control). However, against strain VR-733, oral PDSTP at 80 mg/kg/day protected 30–40% of animals and extended mean lifespan approximately 1.9-fold compared to untreated controls (*p* < 0.05), comparable to acyclovir at 100 mg/kg/day (40% survival). IP PDSTP at 25 mg/kg/day provided 50% survival in the VR-733 IN model, matching the efficacy of acyclovir at 10 mg/kg/day. These results indicate that PDSTP monotherapy is active against HSV-1 in vivo, but its efficacy is strain-dependent and dose-dependent.

A consistent finding across all experiments was the markedly greater efficacy of IP versus PO administration. For example, in the VR-733 IP infection model, PO PDSTP at 80 mg/kg/day yielded 30–40% survival, whereas IP PDSTP at 25 mg/kg/day achieved 50% survival, despite the latter being administered at a substantially lower dose. Similarly, in combination therapy, PO PDSTP (50 mg/kg/day) + PO acyclovir (50 mg/kg/day) protected 55% of animals, while IP PDSTP (12.5 mg/kg/day) + IP acyclovir (10 mg/kg/day) achieved 100% survival dramatically lower total drug dose yet superior outcome. This difference is statistically significant (*p* < 0.01 for IP combination vs. PO combination). The most plausible explanation for this is pharmacokinetic. PDSTP is a highly hydrophilic, cationic molecule bearing quaternary nitrogen atoms, which are known to limit passive diffusion across biological membranes [13,15]. The observation that higher PDSTP doses did not consistently improve efficacy, and in some cases appeared less effective than lower doses, deserves comment. This dose–response pattern may reflect saturation of HSPG binding sites, where the excess compound provides no additional antiviral benefit, or may indicate a narrow therapeutic window [13]. Similarly, reduced efficacy and signs of toxicity were reported when the dose of a related dispirotripiperazine compound was increased beyond 70 mg/kg/day [13]. Our data suggest that careful dose optimization is essential for clinical development of PDSTP and related compounds. The complete suppression of brain viral load achieved with IP combination therapy in the IN infection model is particularly noteworthy. Complete protection and suppression of virus replication in the brain were demonstrated in the intranasal VR-733 model.

Study Limitations. Several limitations should be acknowledged. First, the preventive–therapeutic regimen included a prophylactic dose (2 h pre-infection), which does not reflect the typical clinical scenario of delayed diagnosis in HSE patients. However, this design was chosen to establish proof-of-concept and to ensure that any antiviral effect was not merely due to post-infection mitigation of already established infection. Second, only female mice were used, limiting generalizability, though this was justified. The choice of female mice is determined by the need to ensure reproducibility of the results, minimize data variability, and comply with generally accepted modeling protocols for herpetic encephalitis. Despite these limitations, the consistency of our findings across two infection models, two viral strains, two routes of administration, and multiple efficacy endpoints (survival, weight loss, brain viral titer) provides robust evidence for the antiviral potential of PDSTP, particularly in combination with acyclovir.

While these results are promising, they were obtained using a prophylactic–therapeutic regimen and do not directly model the clinical scenario of treatment initiated after disease onset. Future studies are warranted to evaluate PDSTP efficacy in a strictly therapeutic setting.

## 5. Conclusions

This study provides an in vivo evaluation of PDSTP, a novel dispirotripiperazine-based inhibitor targeting HSPG-mediated viral attachment, in mouse models of HSV-1 encephalitis. Viral infection was induced via intraperitoneal or intranasal inoculation, and PDSTP was administered either orally or intraperitoneally. Our results demonstrate dose- and strain-dependent antiviral activity of PDSTP as monotherapy, and, more importantly, a marked enhancement of therapeutic outcomes when PDSTP is combined with acyclovir. Oral administration of PDSTP at 80 mg/kg/day resulted in 30–40% survival in mice infected with HSV-1 strain VR-733 and increased the mean survival time 1.9-fold compared to the untreated control group (*p* < 0.05), which was comparable to the effect of acyclovir at 100 mg/kg/day. IP administration was substantially more effective than oral delivery: PDSTP at 25 mg/kg/day achieved 50% survival in VR-733-infected mice, compared to 30–40% with PO PDSTP at 80 mg/kg/day, despite the more-than-three-fold-lower dose. The most notable finding was the pronounced efficacy of combination therapy: co-administration of PDSTP (12.5 mg/kg/day) and acyclovir (10 mg/kg/day) via the IP route resulted in 100% survival, significant attenuation of weight loss, and a substantial reduction in brain viral load (*p* < 0.001). This effect was achieved at relatively low individual doses, underscoring the therapeutic potential of this combination. The observed enhancement is mechanistically plausible: PDSTP inhibits viral attachment to host cell heparan sulfate proteoglycans, thereby reducing the number of virions capable of entering cells, while acyclovir selectively inhibits viral DNA replication within already infected cells. The lack of a consistent dose–response relationship at higher PDSTP doses likely reflects saturation of HSPG binding sites or a narrow therapeutic window, highlighting the importance of dose optimization rather than maximizing dosage. Although the study has limitations—including the inclusion of a pre-infection (prophylactic) preventive–therapeutic component that does not fully reflect typical clinical scenarios, and the use of female mice only—the consistency of results across two infection models, two HSV-1 strains, two administration routes, and multiple efficacy endpoints supports the antiviral potential of PDSTP. In conclusion, PDSTP represents a promising candidate for further preclinical development, particularly in combination with acyclovir, as this approach may allow for reduced acyclovir doses and potentially overcome challenges associated with drug-resistant HSV strains. Given that mortality from HSE remains approximately 20% and neurological sequelae are substantial despite current standard therapy, improved preventive–therapeutic strategies are urgently needed. Future studies should focus on optimizing PDSTP’s pharmacokinetic profile to enhance oral bioavailability, evaluating activity against acyclovir-resistant HSV strains, developing therapeutic (post-infection) and preventive–therapeutic regimens, and conducting comprehensive safety assessments to define the therapeutic window.

## Data Availability

The original contributions presented in this study are included in the article/Supplementary Material. Further inquiries can be directed to the corresponding author(s).

## Supplementary Materials

The following supporting information can be downloaded at https://www.mdpi.com/article/10.3390/v18090944/s1, Table S1: Efficacy of the PDSTP substance in a mouse model of herpetic encephalitis with intraperitoneal infection with HSV-1; Table S2: Efficacy of the PDSTP substance in a mouse model of herpetic encephalitis in case of intraperitoneal infection with HSV-1 strain VR-539 during oral and intraperitoneal treatment; Table S3: Efficacy of the PDSTP substance in a mouse model of herpetic encephalitis in case of intranasal infection with HSV-1 strain VR-539 during oral treatment; Table S4: Efficacy of PDSTP in a mouse model of herpetic encephalitis in intranasal HSV-1 infection with oral and intraperitoneal treatment; Table S5: The effect of oral and intraperitoneal treatment with the substance alone and in combination with Acyclovir on the titer of HSV-1 strain in the brain of mice with intraperitoneal infection; Table S6: The effect of oral and intraperitoneal treatment with the substance alone and in combination with Acyclovir on the HSV-1 strain VR-733 titer in the brain of mice during their intranasal infection.
