# Supplementary material for: A Proof-of-Concept Study for the Efficacy of Dispirotripiperazine PDSTP in a Mouse Model of Herpes Simplex Herpetic Encephalitis
Source: Viruses. 2026 Aug 28;18(9):944. doi: 10.3390/v18090944 (PMC13611870; doi:10.3390/v18090944)
Supplement: Supplementary file 1 [file viruses-18-00944-s001.zip › viruses-4484700-supplementary.pdf]

Supplementary materials

A PROOF-OF-CONCEPT STUDY FOR THE EFFICACY OF DISPIROTRIPIPERAZINE PDSTP IN A MOUSE MODEL OF HERPES SIMPLEX HERPETIC ENCEPHALITIS

Kristina Pomeshikova [1,2], Olga Riabova [1], Natalia Monakhova [1], Irina Leneva [3], Giuseppina Sanna [4] and Vadim Makarov [1,*]

[1] Federal Research Centre "Fundamentals of Biotechnology" of the Russian Academy of Sciences (Research Centre of Biotechnology RAS), 33-2 Leninsky Prospect, Moscow 119071, Russia; k.pomeshikova@fbras.ru (K.P.); o.riabova@fbras.ru (O.R.); n.monakhova@fbrsa.ru (N.M.)

[2] I.M. Sechenov First Moscow State Medical University, Moscow 119991, Russia

[3] Department of Virology, Mechnikov Research Institute of Vaccines and Sera, Moscow 105064, Russia; wnyfd385@yandex.ru

[4] Microbiology and Virology Unit, Department of Biomedical Science, University of Cagliari, 09042 Monserrato, Italy; g.sanna@unica.it

* Correspondence: author: makarov@inbi.ras.ru

* Corresponding author

E-mail address: makarov@inbi.ras.ru (V.A. Makarov)

Table S1. Efficacy of the PDSTP substance in a mouse model of herpetic encephalitis with intraperitoneal infection with HSV-1.

Drug dose	**Strain VR-539**				**Strain VR-733**			
	Survival rate		Mortality protection rate (%)	Mean day to death(days)	Survival rate		Mortality protection rate (%)	Mean day to death Average life expectancy (days)
	alive/total	% of mortality			alive/total	% of lethality		
**PDSTP substance** (oral treatment)								
40 mg/kg/day<br>80 mg/kg/day	0/10<br>0/11	100<br>100	0<br>0	**7.5d.**(1-7d.,3-8d.,3-9d.,3-10d.)<br>**5.1d.** (2-3d., 1-4d., 6-7d.,2-8d.)	1/10<br>3/10	90<br>70	10<br>30	**7.5d.** (1-3d., 3-6d., 3-7d., 2-8d.)<br>**12.1d.** (1-3d., 2-5d.,1-6d.,1-7d.)
**Acyclovir** (oral treatment)								
100 mg/kg/day	0/10	100	0	**6.5d.** (1-3d.,1-4d.,1-5d.,1-6d., 4-7d., 1-8d.,1-9d.)	4/10	60	40	**11.3d.** (2-5d., 3-7d.,1-8d.)
**PDSTP substance** (intraperitoneal treatment)								
10 mg/kg/day<br>20 mg/kg/day<br>50 mg/kg/day<br>70 mg/kg/day	0/10<br>2/10*<br>4/8**<br>0/10	100<br>80<br>50<br>100	0<br>20<br>50<br>0	**5.9d.** (2-3d., 2-5d., 3-6d., 2-7d.)<br>**8.7d.** (1-3d.,1-4d., 3-5d.,2-6d.)<br>**13.75d.** (1-3d., 2-5d.)<br>**6d.** (10-7d.)	1/9<br>2/9<br>6/10<br>0/10	89<br>78<br>40<br>100	11<br>22<br>60<br>0	**6.4d.**(1-3d.,1-4d.,1-5d.,3-6d.,2-8d.)<br>**8d.** (2-4d.,2-5d.,3-7d.)<br>**15.8d.** (1-9d., 3-11d.)<br>**5.1d.** (3-5d.,5-6d.,1-7d., 1-9d.)
**Acyclovir** (intraperitoneal treatment)								
50 mg/kg/day	9/10***	10	90	**18.5d**. (1-6d.)	10/10	0	100	
**Virus control**								
	0/10	100		**6.7d.** (1-3d., 3-5d., 1-6d., 1-7d., 1-9d., 1-11d.,2-13d.)	0/10	100		**6.2d.** (1-4d., 2-5d., 2-6d., 1-7d.,1-8d,2-9d.,1-13d.)

*p<0.05 — Differences are found at the trend level.

**p<0.01 — Statistically significant differences were found.

***p<0.001 — Differences were found at a high level of statistical significance.

Additional figure to Table S1. Effect of treatment with the substance PDSTP on the weight of mice infected intraperitoneally with HSV-1.
A strain of VR-539, oral treatment
B strain VR-733, oral treatment

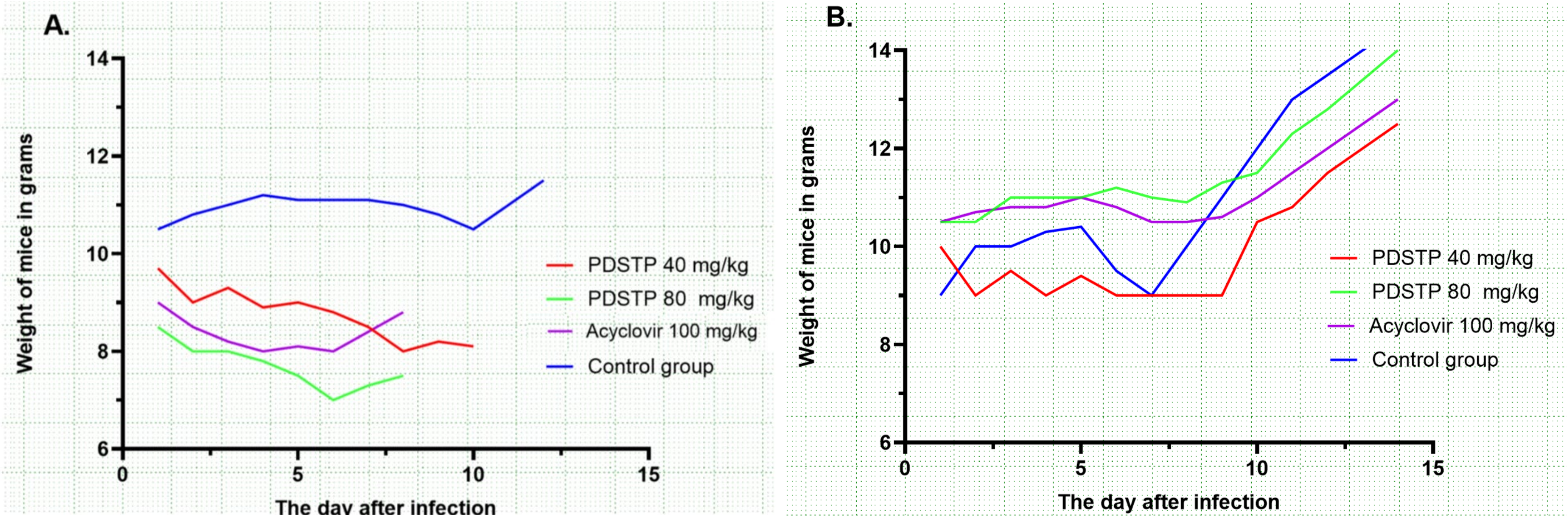

Additional figure to Table S1. The effect of treatment with the substance PDSTP on the weight of mice infected intraperitoneally with HSV-1.
A strain VR-539, intraperitoneal treatment
B strain VR-733, intraperitoneal treatment

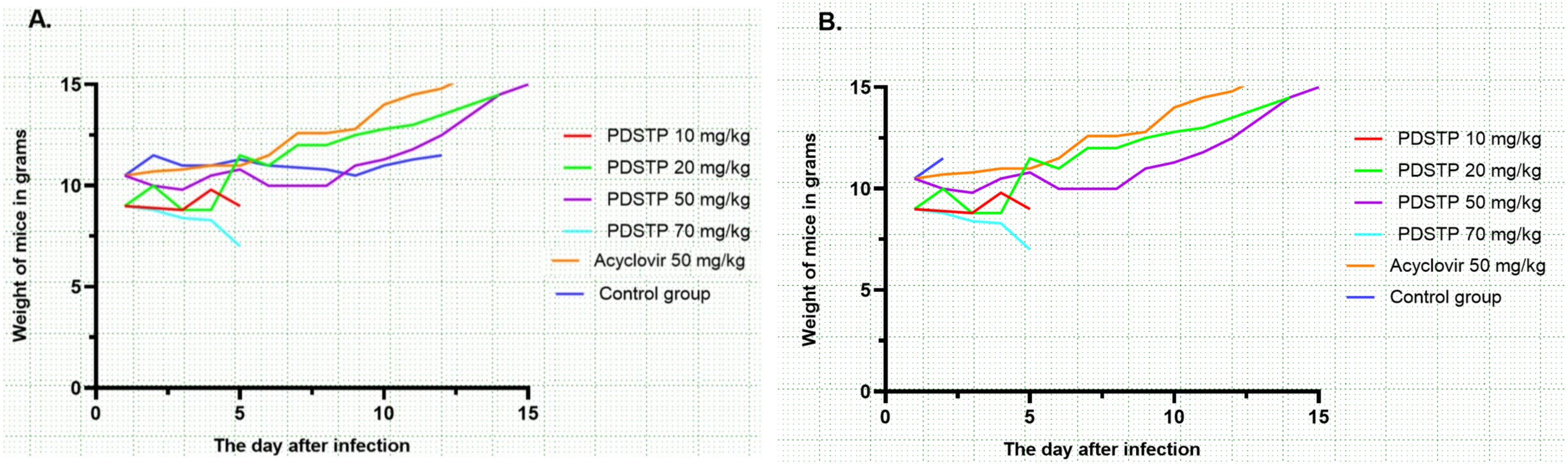

Table S2. Efficacy of the PDSTP substance in a mouse model of herpetic encephalitis in case of intraperitoneal infection with HSV-1 strain VR-539 during oral and intraperitoneal treatment.

Drug dose	**Strain VR-539**			
	Survival rate		Mortality protection rate (%)	Average life expectancy (days)
	alive/total	% of mortality		
**PDSTP substance** (oral treatment)				
50 mg/kg/day<br>70 mg/kg/day	3/9*<br>1/9	67<br>89	33<br>11	**10.4d.** (3-5d.,1-7d.,2-9d.)<br>**6d.** (7-5d, 1-7d.)
**Acyclovir** (oral treatment)				
50 mg/kg/day	0/8	100	0	**4.25d.** (7-5d, 1-7d)
**Acyclovir combination 50 mg/kg/day** (oral treatment)				
PDSTP<br>50 mg/kg/day<br>70 mg/kg/day	 5/9**<br>1/8	 45<br>88	 55<br>12	 **14.1d.** (1-5d.,1-8d., 2-9d.)<br>**8.5d.** (4-7d, 2-8d., 1-11d.)
**PDSTP substance** (intraperitoneal treatment)				
12.5 mg/kg/day<br>25 mg/kg/day	0/10<br>0/10	100<br>100	0<br>0	**3.6d.** (2-3d, 8-5d.)<br>**2.2d.** (9-3d, 1-5d.)
**Acyclovir** (intraperitoneal treatment)				
10 mg/kg/day	1/9	89	11	**4.9d.** (8-4d.)
**Acyclovir combination 10 mg/kg/day** (intraperitoneal treatment)				
PDSTP<br>12.5 mg/kg/day<br>25 mg/kg/day	 10/10***<br>5/10**	 0<br>50	 100<br>50	 **>15d.**<br>**12.7d.** (3-5d.,1-8d., 1-9d.)
**Virus control**				
	0/10	100	0	**6.6d.** (5-5d., 2-9d., 3-11d.)

*p<0.05 — Differences are found at the trend level.

**p<0.01 — Statistically significant differences were found.

***p<0.001 — Differences were found at a high level of statistical significance.

Additional figure to Table S2. (A) Effect of oral treatment with the substance PDSTP on the weight of mice infected intraperitoneally with HSV-1 strain VR-539. (B)The effect of intraperitoneal treatment with PDSTP alone and in combination with Acyclovir on the weight of mice infected with intraperitoneal HSV-1 strain VR-539.

A- oral treatment

B- intraperitoneal treatment

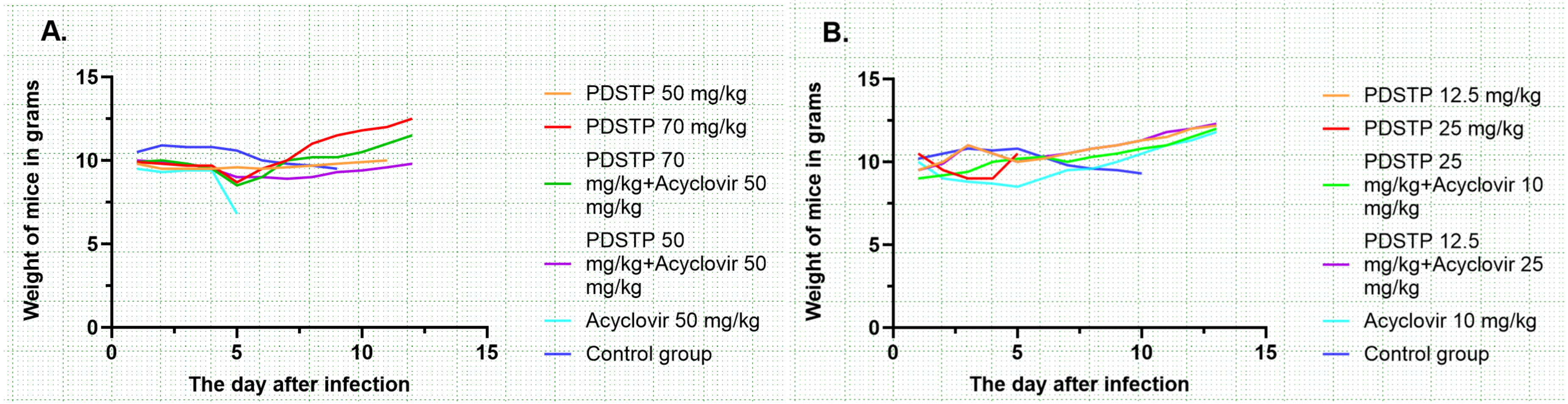

Table S3. Efficacy of the PDSTP substance in a mouse model of herpetic encephalitis in case of intranasal infection with HSV-1 strain VR-539 during oral treatment.

Drug dose	**Strain VR-539**			
	Survival rate		Mortality protection rate (%)	Average life expectancy (days)
	alive/total	Mortality protection rate (%)		
**PDSTP substance**				
10 mg/kg/day<br>25 mg/kg/day<br>50 mg/kg/day	0/10<br>2/10*<br>0/10	100<br>80<br>100	0<br>20<br>0	**5.8d.** (1-4d, 2-5d.,5-6d.,1-9d.,1-10d.)<br>**8.9d.** (2-6d, 3-7d.,3-8d.)<br>**5.8d**. (2-6d., 8-7d.)
**Acyclovir**				
50 mg/kg/day<br>100 mg/kg/day	4/10*<br>8/10***	60<br>20	40<br>80	**12d.** (2-6d., 1 -7d., 3-9d.)<br>**17.5d.** (1-8d,1-9d.)
**Virus control**				
	0/10	100		**5.5d.** (1-3d, 1-4d.,1-6d.,4-7d.,3-8d.)

*p<0.05 — Differences are found at the trend level.

**p<0.01 — Statistically significant differences were found.

***p<0.001 — Differences were found at a high level of statistical significance.

Table S4. Efficacy of PDSTP in a mouse model of herpetic encephalitis in intranasal HSV-1 infection with oral and intraperitoneal treatment.

Preparation	Survival rate		Index Mortality Protection (%)	Average lifetime (days)
	Alive/ Total	% of mortality		
**PDSTP substance** (oral treatment)				
25 mg/kg/day	0/10	100	0	**5d.** (1-4d, 2-5d.,5-6d.,1-7d.,1-9d.)
40 mg/kg/day	2/10*	80	20	**8.9d.** (2-6d, 3-7d., 3-8d.)
50 mg/kg/day	3/10*	70	30	**10.6d.** (4-7d, 2-8d, 1-9d.)
75 mg/kg/day	1/9	90	10	**7.9d.** (4-7d, 5-8d.)
100 mg/kg/day	0/10	100	0	**7.8d.** (2-8 days, 8-9 days)
**Acyclovir** (oral treatment)				
50 mg/kg/day	4/9*	56	44	**12.7d.** (2-8d, 3-12d)
**Combination of Acyclovir 50 mg/kg/day** (oral treatment) + **PDSTP substance**				
50 mg/kg/day	9/10***	10	90	**19d.** (1-11d.)
75 mg/kg/day	4/10*	60	40	**12.9d.** (2-8d., 2-9d.,1-10d.,1-11d.)
**PDSTP Substance** (Intraperitoneal Treatment)				
12.5 mg/kg/day	0/10	100	0	**5.6d.** (2-4d, 1-6d, 4-7d., 3-8d.)
25 mg/kg/day	6/12**	50	50	**16.3d.** (1-6d, 3-7d, 2-11d)
40 mg/kg/day	1/9	90	11	**4.8d.** (2-4d.,2-6d.,4-7d., 1-9d.)
**Acyclovir** (intraperitoneal treatment)				
10 mg/kg/day	5/10**	50	50	**13.6d.** (3-7d., 1-9d., 1-11d.)
**Combination of Acyclovir 25 mg/kg/day** (treatment intraperitoneally) + **PDSTP substance**				
12.5 mg/kg/day	10/10***	0	100	**>15d.**
25 mg/kg/day	10/10***	0	100	**>15d.**
**Virus Control**				
	0/10			**7.4d.** (7-7d., 2-11d, 1-13d.)

*p<0.05 — Differences are found at the trend level.

**p<0.01 — Statistically significant differences were found.

***p<0.001 — Differences were found at a high level of statistical significance.

Table S5. The effect of oral and intraperitoneal treatment with the substance alone and in combination with Acyclovir on the titer of HSV-1 strain in the brain of mice with intraperitoneal infection.

Drug dose	Mortality protection rate (%)	Virus titer in the brain ($TCID_{50}$)
**PDSTP substance** (oral treatment)		
50 mg/kg/day<br>70 mg/kg/day	33<br>11	$3.16×10^3$ ($1.0×10^3$ to $1.0×10^4$)<br>$3.72×10^1$ ($1.48×10^1$ to $9.33×10^1$)
**Acyclovir** (oral treatment)		
50 mg/kg/day	0	$1.0×10^3$ ($3.16×10^2$ to $3.16×10^3$)
**Combination of Acyclovir 50 mg/kg/day** (oral treatment)+		
PDSTP substance<br>50 mg/kg/day<br>70 mg/kg/day	 55<br>12	 $1.0×10^1$ ($5.50×10^0$ to $1.82×10^1$)<br>$1.0×10^0$ ($3.16×10^0$ to $3.16×10^1$)
**PDSTP substance** (intraperitoneal treatment**)**		
12.5 mg/kg/day<br>25 mg/kg/day	0<br>0	$1.17×10^4$ ($9.12×10^3$ to $1.51×10^4$)<br>$2.14×10^2$ ($5.75×10^1$ to $7.94×10^2$)
**Acyclovir** (intraperitoneal treatment)		
10 mg/kg/day	11	$2.69×10^3$ ($9.33×10^2$ to $7.76×10^3$)
**Combination of Acyclovir 50 mg/kg/day** (intraperitoneal treatment)+		
PDSTP substance<br>12.5 mg/kg/day<br>25 mg/kg/day	 100<br>50	 $2.51×10^1$ ($1.70×10^1$ to $3.72×10^1$)<br>$1.0×10^0$
**Virus control**		$6.76×10^4$ ($3.55×10^4$ to $1.29×10^5$)

Table S6. The effect of oral and intraperitoneal treatment with the substance alone and in combination with Acyclovir on the HSV-1 strain VR-733 titer in the brain of mice during their intranasal infection.

Drug dose	Mortality protection rate (%)	Virus titer in the brain ($TCID_{50}$)
**PDSTP substance** (oral treatment)		
40 mg/kg/day<br>50 mg/kg/day<br>75 mg/kg/day	20<br>30<br>10	2.51×10^{1} (1.70×10^{1} to 3.72×10^{1})<br>1.45×10^{1} (7.41×10^{0} to 2.82×10^{1})<br>6.67×10^{0} (2.04×10^{0} to 2.24×10^{1})
**Acyclovir** (oral treatment)		
50 mg/kg/day	44	2.14×10^{0} (1.10×10^{0} to 4.17×10^{0})
**Combination of Acyclovir 50 mg/kg/day** (oral treatment)+		
PDSTP substance<br>50 mg/kg/day<br>75 mg/kg/day	 90<br>10	 1.20×10^{0} (8.71×10^{-1} to 1.66×10^{0})<br>1.0×10^{0}
**PDSTP substance** (intraperitoneal treatment)		
12.5 mg/kg/day<br>25 mg/kg/day	0<br>50	2.63×10^{0} (7.94×10^{-1} to 8.71×10^{0})<br>1.20×10^{0} (8.71×10^{-1} to 1.66×10^{0})
**Acyclovir** (intraperitoneal treatment)		
10 mg/kg/day	50	1.20×10^{0} (8.71×10^{-1} to 1.66×10^{0})
**Combination of Acyclovir 50 mg/kg/day** (intraperitoneal treatment)+		
PDSTP substance<br>12.5 mg/kg/day<br>25 mg/kg/day	 100<br>100	 1.0×10^{0}<br>1.0×10^{0}

**Virus control**		2.14×10^2 (5.75×10^1 to 7.94×10^2)
